# Direct analysis of mAb aggregates in mammalian cell culture supernatant

**DOI:** 10.1186/s12896-014-0099-3

**Published:** 2014-11-29

**Authors:** Albert J Paul, Karen Schwab, Friedemann Hesse

**Affiliations:** Institute of Applied Biotechnology (IAB), Biberach University of Applied Sciences, 88400 Biberach, Germany

**Keywords:** Protein aggregation, Monoclonal antibodies, Mammalian cell culture, CHO cells

## Abstract

**Background:**

Protein aggregation during monoclonal antibody (mAb) production can occur in upstream and downstream processing (DSP). Current methods to determine aggregate formation during cell culture include size exclusion chromatography (SEC) with a previous affinity chromatography step in order to remove disturbing cell culture components. The pre-purification step itself can already influence protein aggregation and therefore does not necessarily reflect the real aggregate content present in cell culture. To analyze mAb aggregate formation directly in the supernatant of Chinese hamster ovary (CHO) cell culture, we established a protocol, which allows aggregate quantification using SEC, without a falsifying pre-purification step.

**Results:**

The use of a 3 μm silica SEC column or a SEC column tailored for mAb aggregate analysis allows the separation of mAb monomer and aggregates from disturbing cell culture components, which enables aggregate determination directly in the supernatant. Antibody aggregate analysis of a mAb-producing CHO DG44 cell line demonstrated the feasibility of the method. Astonishingly, the supernatant of the CHO cells consisted of over 75% mAb dimer and larger oligomers, representing a substantially higher aggregate content than reported in literature so far.

**Conclusion:**

This study highlights that aggregate quantification directly in the cell culture supernatant using appropriate SEC columns with suitable mAb aggregate standards is feasible without falsification by previous affinity chromatography. Moreover, our results indicate that aggregate formation should be addressed directly in the cell culture and is not only a problem in DSP.

**Electronic supplementary material:**

The online version of this article (doi:10.1186/s12896-014-0099-3) contains supplementary material, which is available to authorized users.

## Background

Over the past ten years, the demand for monoclonal antibodies (mAbs) as biopharmaceutical drugs for the treatment of cancer and other diseases has increased [1-3]. Like other recombinant therapeutic proteins, mAbs are mainly produced in mammalian cells, usually in Chinese hamster ovary (CHO) cells [4]. Antibody manufacturing includes several steps, where environmental factors such as pH, temperature, ionic strength, protein concentration, oxygen and shear forces can lead to aggregate formation during upstream (USP) and downstream (DSP) processing [5,6]. Kramarcyk et al. reported up to 20-30% aggregate content of a partially purified mAb produced in CHO cells [7]. Self-association and formation of aggregates are a major concern for therapeutic applications, since aggregates influence drug performance and safety [8,9].

DSP offers the opportunity to remove aggregates, but this often leads to a reduction of protein yields. Another strategy involves reducing the formation of aggregates in the cell culture [10]. Jing and colleagues showed that proper control of culture conditions during USP successfully reduced the level of protein aggregation and improved the process yield [11]. To evaluate aggregate formation upstream, suitable analytical methods for aggregate detection and quantification are essential. This is challenging, since the size of aggregates may range from small oligomers to visible particles. Furthermore, host cell proteins (HCPs) and cell culture medium components may complicate aggregate detection. Nowadays, investigation of protein aggregation during cell cultivation is usually performed after a Protein A capture step [11-14]. Protein A affinity chromatography is a powerful tool for antibody purification, but it also exposes antibodies to pH-shifts [15]. This pH shift favors the formation of aggregates, since the aggregation rate of proteins is strongly influenced by pH [16,17]. Phillips et al. showed that acidic pH values of Protein A elution led to substantial protein aggregation and precipitation [18]. This implies that Protein A purification itself might influence the aggregation status, thus purified samples do not necessarily reflect the aggregation state of mAbs in cell culture.

Size exclusion chromatography (SEC) used in a high pressure liquid chromatography (HPLC) system is the most commonly applied analytical method for the analysis of soluble protein aggregates [19]. Typically, SEC analysis is performed after Protein A chromatography-based isolation of the mAbs from the cell culture supernatant, because cell culture components interfere with the direct analysis of mAb cell culture samples on SEC columns [10]. In the present study, we developed a procedure to analyze aggregate formation directly in the supernatant of CHO cells without a pre-purification step. Using a SEC column consisting of 3 μm silica particles or a SEC column especially tailored for mAb analysis due to selected pore size and column dimensions, we were able to separate mAb monomer and aggregates from interfering signals caused by DNA, host cell and culture medium components. The high resolution of these columns enabled quantification of mAb monomer and aggregate content directly from cell culture samples. In order to distinguish between the aggregates formed in cell culture, mAb aggregates differing in size and morphology were induced using different stress methods. The different high molecular weight (HMW) species were identified as dimers, tetramers, oligomers and large particles with hydrodynamic diameters greater than 100 nm. Additionally, stability and traceability of the induced mAb aggregates under conditions similar to the cell culture environment were shown. The induced aggregates served as standards for aggregate analysis in the culture supernatant. Finally, we analyzed aggregate formation of a mAb-producing CHO DG44 cell line to demonstrate that monomer and aggregates were detectable and quantifiable directly in the supernatant without a pre-purification step.

## Methods

### Monoclonal antibodies, cell line and media

Two aggregation-prone monoclonal antibodies (mAb1 and mAb2) were used as model proteins for this study. The mAbs were produced in CHO DG44 cells [20,21]. The cells were cultivated in defined protein-free medium (SFM4CHO, Thermo Scientific). A non-producing CHO DG44 cell line was used as control, which was supplemented with hypoxanthine and thymidine (HT). The antibodies were Protein A purified, filtered (0.2 μm) and stored in 20 mmol L^−1^ acetate at pH 3.5 for further studies. After stress induction the samples were filtered (0.2 μm) prior to SE-HPLC analysis.

### Generation of different mAb aggregates

A pH-shift, high-salt concentration or freeze-thawing (FT) were used to induce different types of mAb aggregates. The antibodies were diluted to a final concentration of 1 mg mL^−1^ for the aggregation studies and analyzed immediately after stress-induction.

In order to generate pH-dependent aggregation, the mAbs were diluted in 100 mmol L^−1^ citric acid (2-hydroxypropane-1,2,3-tricarboxylic acid) and 200 mmol L^−1^ disodium hydrogen phosphate (Na_2_HPO_4_) with pH values ranging from pH 3–8. For exposure to high salt concentrations the mAbs were diluted to final sodium chloride (NaCl) concentrations ranging from 0.1 to 1.5 mol L^−1^. For this purpose, a 5 mol L^−1^ NaCl stock solution was prepared using ultrapure water (Millipore). Moreover, both antibodies were subjected to multiple FT cycles. One FT cycle included storage at −80°C for 15 min, followed by thawing at 25°C for 15 min in a thermal mixer (HLC Biotech).

### SE-HPLC analysis

Analysis of soluble aggregates was performed using an Agilent 1100 HPLC (Agilent Technologies) system and an UltiMate 3000 (Thermo Scientific) system. TSKgel G3000SWXL (Tosoh Bioscience), Yarra SEC-4000 (Phenomenex) and MAbPac SEC-1 (Thermo Scientific) were used as SEC columns. The column properties are listed in the Additional file 1: Table S1. The SEC separation was performed at ambient temperature isocratically using a mobile phase consisting of phosphate-buffered saline (PBS), which was adjusted to pH 7.2 and filtered 0.1 μm prior to use. Flow rates varied between 0.3, 0.5 and 0.8 mL min^−1^. The respective amounts of mAb monomer, aggregates and fragments were quantified by calculation of the peak areas detected by the ultraviolet (UV) detectors. The UV signal of the Agilent 1100 system was displayed in mV, the signal of the Ultimate 3000 system in mAU. All samples were pre-filtered using 0.2 μm syringe filters (Phenomenex).

### Dynamic light scattering (DLS)

Formation of large aggregates was determined using a Zetasizer 3000 HS instrument (Malvern Instruments) at 25°C. The average hydrodynamic diameter (Zave) of the HMW species was measured in disposable semi-micro UV cuvettes (Brand).

### Molecular weight determination

The molecular weight of the aggregates was determined using SEC in combination with multi-angle light scattering (MALS). SEC-MALS was performed using an Äkta Explorer 100 instrument (GE Healthcare) equipped with a Yarra SEC-4000 column (Phenomenex), an Optilab T-Rex refractive index (RI) detector (Wyatt) and a DAWN HELEOS 8+ MALS detector (Wyatt). The samples were analyzed at room temperature (RT) using 0.1 μm filtered PBS (pH 7.0).

### Stability of the induced aggregates

To investigate aggregate stability, mAb2 was stressed using 500 mmol L^−1^ NaCl or three cycles of FT, respectively, in order to induce dimer and oligomer formation and measured after storage at RT for up to 72 h. To investigate the traceability under cell culture conditions, stressed antibody samples were spiked into SFM4CHO medium or CHO DG44 host cell supernatant and analyzed immediately afterwards.

### Aggregate formation in mAb-producing CHO cell line

To investigate aggregate formation in cell culture, CHO DG44 cells producing mAb2 were analyzed directly after inoculation and after 144 h of cultivation, respectively. For this purpose, cells were seeded at an initial concentration of 4 × 10^5^ mL^−1^ in 125 mL shake flasks (Corning) with a working volume of 25 mL. Cultivation was performed in SFM4CHO medium supplemented with 10 g L^−1^ glucose and 4 mmol L^−1^ glutamine at 37°C, 140 rpm, 80% humidity and 5% CO_2_. For analysis of mAb aggregate content using SE-HPLC, cells were separated at 10.000 g for 5 min and the supernatant was filtered (0.2 μm).

### Determination of DNA and host cell proteins

To ensure the absence of DNA and HCPs in the SEC fractions used for aggregate quantification, the corresponding fractions of the CHO mAb2 supernatant were collected (fraction volume: 98 μL) and analyzed. For DNA quantification, the fractions were analyzed using a NanoDrop 1000 spectrophotometer (Thermo Scientific). For the determination of HCP and culture medium components, the SEC fractions were separated using sodium dodecyl sulfate polyacrylamide gel electrophoresis (SDS-PAGE) and protein bands were detected using silver staining. To receive sufficient amounts for analysis the SEC fractions were pooled and concentrated. Concentration was performed using Vivaspin 500 with a 3 kDa molecular weight cut-off (Sartorius Stedim Biotech). SDS-PAGE was performed at 160 V for 70 min at RT under reducing conditions with an Amersham ECL Gel Box and a 4-20% Amersham ECL Gel (GE Healthcare). Purified mAb2 and supernatant of non-producing CHO cells served as controls. PageRuler Prestained Protein Ladder (Thermo Scientific) was used as size standard.

## Results and discussion

### SEC-based analysis of mAb aggregates in cell culture supernatant

**Figure 1:**
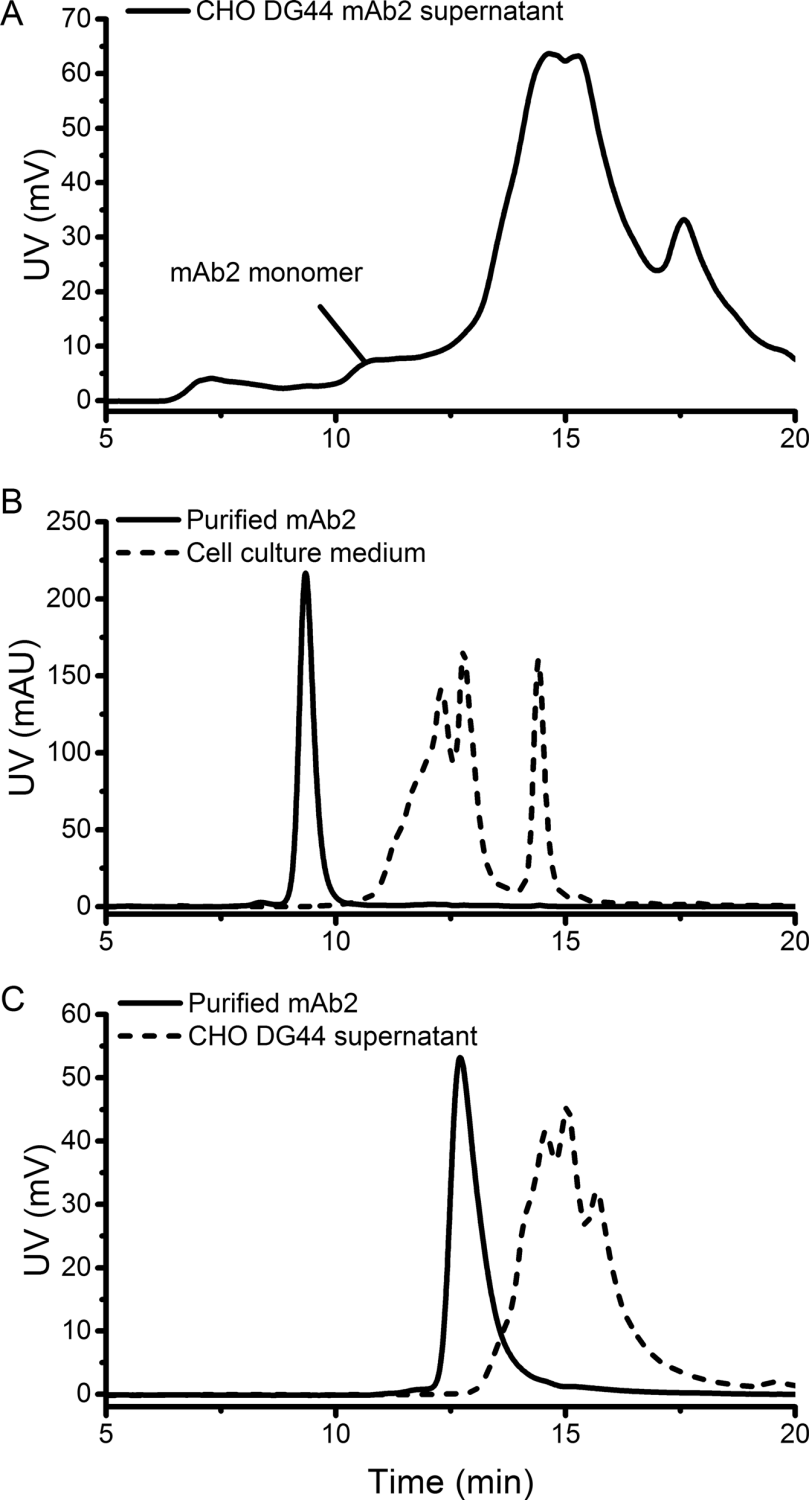
**SEC analysis of mAb2 cell culture samples.** CHO cell culture supernatant analyzed using a common SEC (TSKgel G3000SWXL) column **(A)**. Analysis of purified mAb2 compared to SFM4CHO medium using MAbPac SEC-1 **(B)** and CHO DG44 supernatant using Yarra SEC-4000 **(C)**.

MAbs produced in mammalian cells are secreted into the cell culture supernatant, where formation of aggregates can be detected using SEC analysis after a Protein A capture step [4,15]. This approach is time-consuming, laborious and can critically influence the aggregation status. Direct analysis of cell culture samples containing mAb using SEC is complicated, since components such as DNA, lipids and HCPs can interfere with the analysis of the product [10]. Accordingly, analysis of CHO mAb2 fermentation supernatant samples using a common SEC column (TSKgel G3000SWXL) was insufficient and therefore confirmed that separation of mAb monomer from other supernatant signals disturbed aggregate detection (Figure 1A). Since analysis of cell culture samples using SEC appeared to be a problem of separation capacity, columns promising higher separation efficiencies were tested. Using a SEC column specifically designed for mAb analysis (MAbPac SEC-1) showed that cell culture medium components eluted later than purified mAb monomer (Figure 1B). Serum-free media often contain growth factors, lipoproteins and other factors as growth-promoting supplements. It was confirmed that none of the media components interfered with the analysis of the mAb2 monomer as these components showed higher retention times. Furthermore, analysis of the supernatant from the CHO DG44 culture using a 3 μm SEC column (Yarra SEC-4000) revealed that also the cell culture components in the supernatant eluted later than the mAb monomer (Figure 1C). These host cell components, i.e. cellular proteins, DNA and other components usually complicate the detection of target proteins [22]. Our results indicated that cell culture and medium impurities eluted later than the mAb monomer using columns providing sufficiently high separation efficiency. Therefore, both columns were tested for their applicability for the analysis of mAb aggregates in cell culture samples.

### Generation of different mAb aggregates

For aggregate detection during cultivation, one need to be capable of measuring and characterizing all types of aggregates potentially present in the culture broth. Therefore, different types of mAb aggregates were generated by applying different stress conditions. Since pH changes and increase of osmolality are both known phenomena occurring in cell culture, pH-shift and high salt concentration were used as induction methods. Furthermore, to generate high amounts of soluble aggregates, FT was additionally used as unnatural stress condition. To distinguish between the different HMW species, the molecular weight was determined using SEC-MALS. The various stress methods induced different mAb aggregates, which were used as standards for the analysis of cell culture supernatants.

### pH-shift

As mentioned in the introduction, the pH-shift required for Protein A chromatography favors the formation of aggregates and therefore may lead to reduced antibody yields [17]. Thus, a pH-shift can also be used to induce mAb aggregates. The different antibodies, mAb1 and mAb2, were exposed to different pH values ranging from pH 3–8, which resulted in the formation of small as well as large aggregates (Table 1). With increasing pH a loss of mAb2 monomer was observed accompanied by the formation of mAb1 and mAb2 dimer. At pH 3 mAb1 consisted of only 1.8% dimer and mAb2 of 1.7% dimer, whereas 3.4% and 6.3% dimer were visible at pH 8, respectively. Additionally, a pH shift induced the generation of mAb2 oligomer and a smaller fragment. Oligomer was elevated at pH 3, but less prominent for other pH values. The correlation between pH increase and dimer formation has already been previously described [23]. Ishikawa et al. showed a pH-dependent fragment formation of different antibodies [24]. The antibody used in their study showed most aggregate formation at neutral pH, which is consistent with our results. However, in their work the amount of lower molecular weight species was highest at pH 4, whereas mAb2 formed more fragments at higher pH values. Additionally, DLS revealed a pH-induced formation of large mAb aggregates. With an average diameter greater than 80 nm, the Zave of mAb1 at pH 5 and 6 was more than 2 times higher than at pH 3 (34.2 nm ±1.8 nm) and 4 (32.5 nm ±3.3 nm). MAb2 formed large aggregates at pH 5 with a diameter greater than 100 nm, whereas at other pH values the average diameter was smaller than 50 nm. Since both antibodies formed large aggregates and mAb2 was also degraded to fragments, pH-shift was not used for further stability studies and for the generation of aggregate standards.

**Table 1:** **Formation of mAb dimer, oligomer, fragment and large aggregates using the different induction methods**

	**mAb1**			**mAb2**			
	**Dimer (%)**	**Oligomer (%)**	**Zave (nm)**	**Dimer (%)**	**Oligomer (%)**	**Fragment (%)**	**Zave (nm)**
pH							
3	1.8	-	34.2 ± 1.8	1.7	1.8	-	39.6 ± 2.4
4	1.9	-	32.5 ± 3.3	2.1	0.9	-	49.5 ± 3.5
5	2.3	-	84.3 ± 15.0	2.0	0.9	3.5	144.9 ± 35.8
6	2.6	-	85.7 ± 1.9	3.5	0.6	5.7	47.9 ± 1.3
7	3.1	-	51.1 ± 5.3	4.0	0.5	4.5	45.7 ± 0.8
8	3.4	-	n. a.	6.3	0.6	6.3	n. a.
NaCl							
Control	0.6	-	33.7 ± 5.3	1.1	-	-	33.2 ± 0.2
500 mM	1.3	-	23.8 ± 0.2	3.0	-	-	41.8 ± 1.9
1 M	1.7	-	24.8 ± 0.6	4.2	-	-	56.6 ± 8.8
1.5 M	2.0	-	24.2 ± 1.6	4.6	-	-	78 ± 9.2
FT							
Control	0.6	-	19.7 ± 1.1	1.4	-	-	10.6 ± 1.1
1×	16.0	11.3	20.7 ± 1.8	17.1	3.6	-	14.4 ± 0.1
2×	20.2	16.3	26.6 ± 1.9	19.3	3.9	-	12.9 ± 0.5
3×	22.7	11.0	27.3 ± 2.8	21.0	6.6	-	12.1 ± 0.5

### NaCl

**Figure 2:**
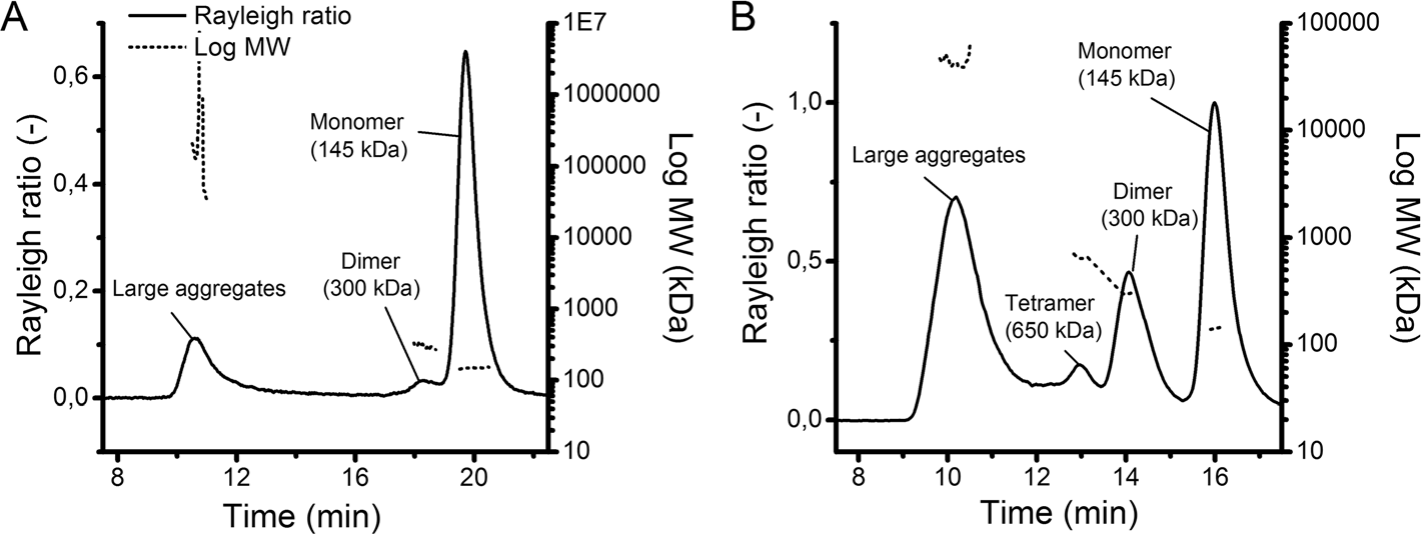
**Molecular weight determination of mAb aggregate standards.** MAb2 aggregate standards were induced using either NaCl **(A)** or FT **(B)** and analyzed using SEC-MALS.

Increased salt concentration reduces the colloidal stability by suppressing electrostatic repulsion, leading to aggregation [25]. We used this mechanism to induce aggregation of the two model proteins. The use of different NaCl concentrations induced the formation of small aggregates as well as large aggregates (Table 1). In comparison to pH-shift induced aggregation, fewer aggregates were formed using NaCl, but it is worth mentioning that NaCl-induction specifically formed one smaller HMW species. Using SEC-MALS, the molecular weight of the NaCl-induced HMW species was determined as a 300 kDa mAb2 dimer (Figure 2A). In addition, the light scattering signal revealed the presence of a large aggregate population, which was not visible using UV detection. With increasing NaCl concentrations mAb1 monomer content decreased and dimer increased from 0.6% (w/o NaCl) to 2.0% (1.5 mol L^−1^ NaCl). Similar to mAb1 also mAb2 monomer decreased with increasing NaCl concentration, whereas the dimer content increased more than fourfold from 1.1% (w/o NaCl) to 4.6% (1.5 mol L^−1^ NaCl). In contrast to mAb2, mAb1 also formed some degradation products. Our results correlate with the results of Fesinmeyer et al., who reported that even in milli-molar concentrations, salts promote mAb aggregate formation [26]. They suggested anion binding as possible reason for aggregation by lowering mAb conformational stability and reduced valence. Analysis with DLS revealed that mAb2 also formed large aggregates (Table 1). NaCl-induction increased the aggregate size up to an average diameter of around 75 nm (1.5 mol L^−1^ NaCl). However, mAb1 did not form large aggregates. Our work showed that increasing ionic strength by 100 mmol L^−1^ NaCl is sufficient to induce mAb dimers, and at concentrations higher than 500 mmol L^−1^ also to induce larger mAb aggregates. Cation concentration in the culture broth increases during the fermentation process due to pH control [27]. Consequently, osmolality in the bioreactor increases over time thus NaCl-induced aggregates are likely to be present in the cell culture broth. Since 500 mmol L^−1^ NaCl induced a significant amount of small aggregates (3.0% dimer) without forming large HMW species of mAb2, this concentration was used to induce mAb2 dimers for stability studies and the generation of aggregate standards.

### Freeze-thawing (FT)

Protein aggregation by FT is attributed to partial unfolding caused by perturbing conditions like low temperature, buffer crystallization, exposure to the ice-liquid interface, adsorption to the container surface, increasing salt or protein concentration [28]. Using FT, both of our model proteins showed the highest aggregation propensity, which illustrated the impact of perturbation on protein stability (Table 1). The aggregates formed by FT were identified as dimer and tetramer (Figure 2B). Again, the light scattering signal showed the presence of a large aggregate population, which was already visible using NaCl-induction, but not measurable using UV detection. Increasing the number of FT cycles resulted in a loss of mAb1 monomer with a corresponding increase of mAb1 dimer (22.7%) and tetramer (11.0%) after three cycles of FT. Similarly, mAb2 comprised around 21.0% dimer and 6.6% tetramer after three cycles of FT. With more than 20% dimer formation, FT induced three times higher aggregate levels than pH-shift to pH 8 (6.3% dimer) and more than four times higher as the highest NaCl concentration (4.6% dimer). Furthermore, FT led to the formation of a significant amount of oligomers (11.0% mAb1, 6.6% mAb2), which were not formed to that extent using the other stress methods. FT also induced formation of large aggregates, which were detectable using DLS. With increasing FT cycle the hydrodynamic diameter of mAb1 aggregates increased from 20 nm to above 25 nm, corresponding to the monomer loss visible in the SEC chromatogram. Interestingly, mAb1 showed the highest transformation rate after three cycles of FT, whereas mAb2 formed most dimer, oligomer and large aggregates after one FT cycle. Our results coincide with Hawe et al., who observed an increase in Zave and the polydispersity index (PdI) caused by FT-induced large and heterogeneous aggregates [29]. Furthermore, our results are also in good accordance with other reports [30]. Since FT induced more aggregates than the other stress methods, this technique was chosen in addition to NaCl-induction for further stability studies and the generation of aggregate standards.

### Stability of the induced aggregates

In order to ensure stability of the aggregate standards, mAb1 and mAb2 were stressed and analyzed after storage at RT. To ensure traceability of the standards in conditions similar to the cell culture environment, the induced aggregates were further analyzed after spiking into cell culture medium and the supernatant of non-producing CHO DG44 cells, respectively.

**Figure 3:**
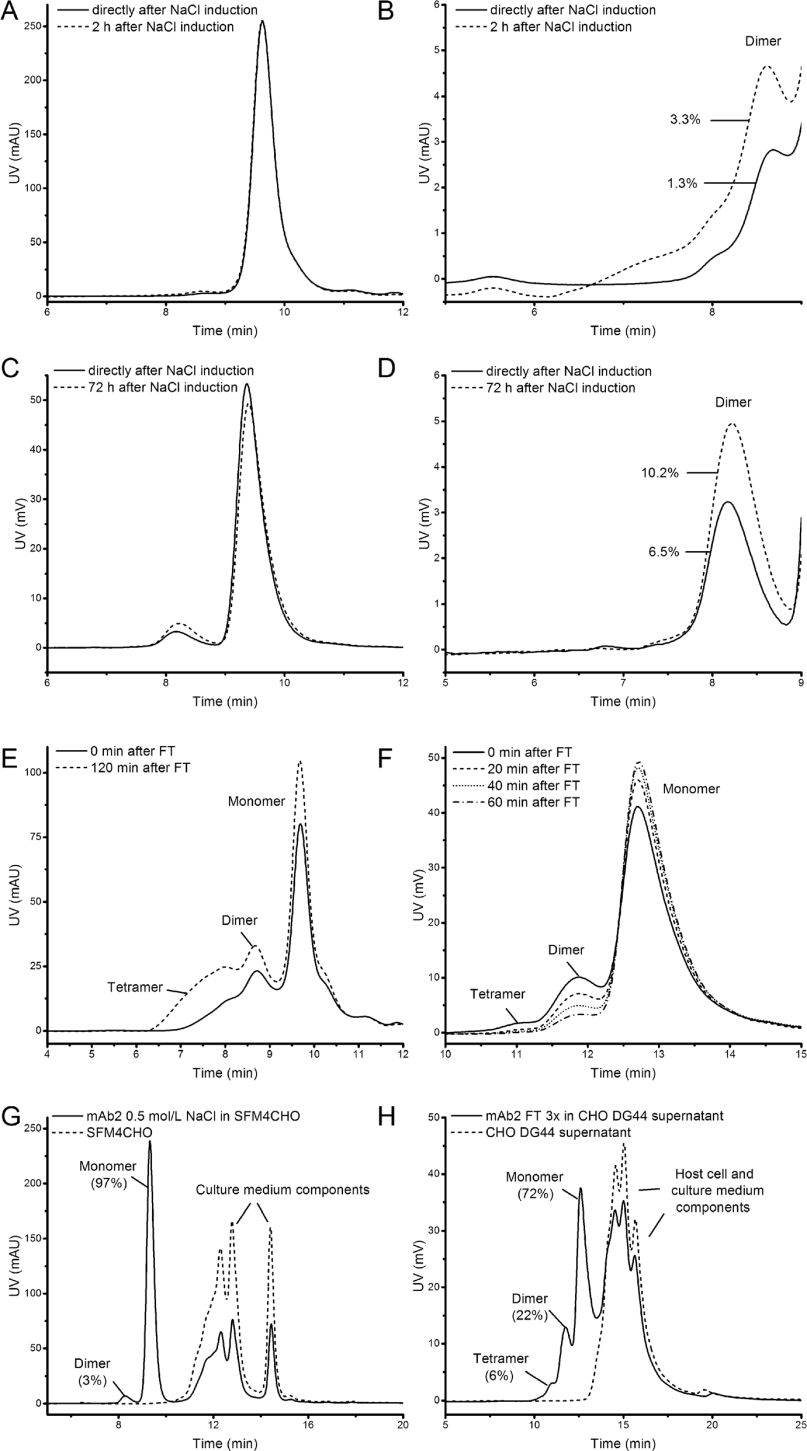
**Stability and traceability of mAb aggregate standards.** The stability of NaCl-induced mAb1 **(A + B)** and mAb2 **(C + D)** aggregates was investigated. **B** is an enlargement of **A**, **D** is an enlargement of **C**. **(E)** Stability of FT-induced mAb1 using MAbPac SEC-1. **(F)** Stability of FT-induced mAb2 aggregates using Yarra SEC-4000. **(G)** Traceability of NaCl-induced mAb2 aggregates using MAbPac SEC-1. **(H)** Traceability of FT-induced mAb2 aggregates using Yarra SEC-4000 under cell culture conditions.

To evaluate stability of NaCl-induced aggregates, mAb1 and mAb2 aggregates were induced using 500 mmol L^−1^ NaCl and analyzed after storage at RT for up to 72 h (Figure 3A-D). MAb1 dimer content increased already after 2 h from 1.3% to 3.3% (Figure 3B) and mAb2 dimer increased within 72 h from 6.5% to 10.2% (Figure 3D), revealing that the aggregation reaction was not finished within this time period. These observations are in good accordance with other reports [26,31,32]. Stability analysis of FT-induced aggregates revealed that the aggregates formed were readily reversible (Figure 3E + F). MAb1 tetramer increased from 11% to 26% after 2 h storage at RT (Figure 3E). FT-induced aggregates of mAb2 seemed to be reversible. Increasing storage time at room temperature resulted in mAb2 monomer recovery with a corresponding decrease in dimer and oligomers (Figure 3F). After 1 h storage at RT, FT-induced mAb2 dimer decreased from 14% to 4% and the tetramer from 2.5% to 0.3%, whereas mAb2 monomer increased from 82.6% to 95.7%. This shift in aggregate distribution was also observed by Philo, who reported that incubation of Protein X at 29°C after FT led to a drop in dimer and larger aggregate content, with a corresponding increase in monomer [33]. Since Philo used Protein X, our results are the first report, to the best of our knowledge, which showed the reversibility of FT-induced aggregates for mAbs. Since the aggregates induced by NaCl and FT were unstable, all aggregate standards were prepared freshly for further experiments.

To study traceability under cell culture conditions, NaCl-induced mAb2 was spiked into SFM4CHO cell culture medium (Figure 3G). Monomer as well as mAb2 aggregates were still detectable in this matrix. Given that the medium components of the serum-free cell culture medium eluted later than mAb2 monomer, quantification of monomer and aggregate content was feasible. With 3% dimer and 97% monomer the values were consistent to NaCl-induced aggregate formation of mAb2 (Table 1). Furthermore, we showed that the dimers and tetramers induced by FT were also detectable and quantifiable in the supernatant of non-producing CHO DG44 cells (Figure 3H). HCPs and culture medium components eluted later than mAb2 monomer and aggregates without influencing aggregate quantification. In total 22% dimer, 6% oligomer and 72% remaining monomer was determined for mAb2, which correlated well to the results obtained from freeze-thawed mAb2 (Table 1). The spiking experiments demonstrated that the aggregates induced by the selected stress methods were stable during the experiment and still detectable under conditions expected in cell culture. Since the high resolution facilitated by the 3 μm SEC column enabled sufficient efficiency to separate mAb monomer and aggregates from CHO DG44 cell culture components, aggregate formation in the culture of a mAb-producing CHO cell line was investigated using this column.

### Aggregate formation in a mAb-producing CHO cell line

To demonstrate the feasibility of the method, the cell-free supernatant derived from a culture of a mAb2-producing CHO cell line was analyzed for aggregate formation. In order to ensure typical growth, culture parameters such as viability and viable cell concentration as well as substrate, metabolite and product concentrations were measured during cultivation. Cell culture samples were taken every 24 h, and aggregation analysis was performed directly after inoculation and after 144 h of cultivation.

**Figure 4:**
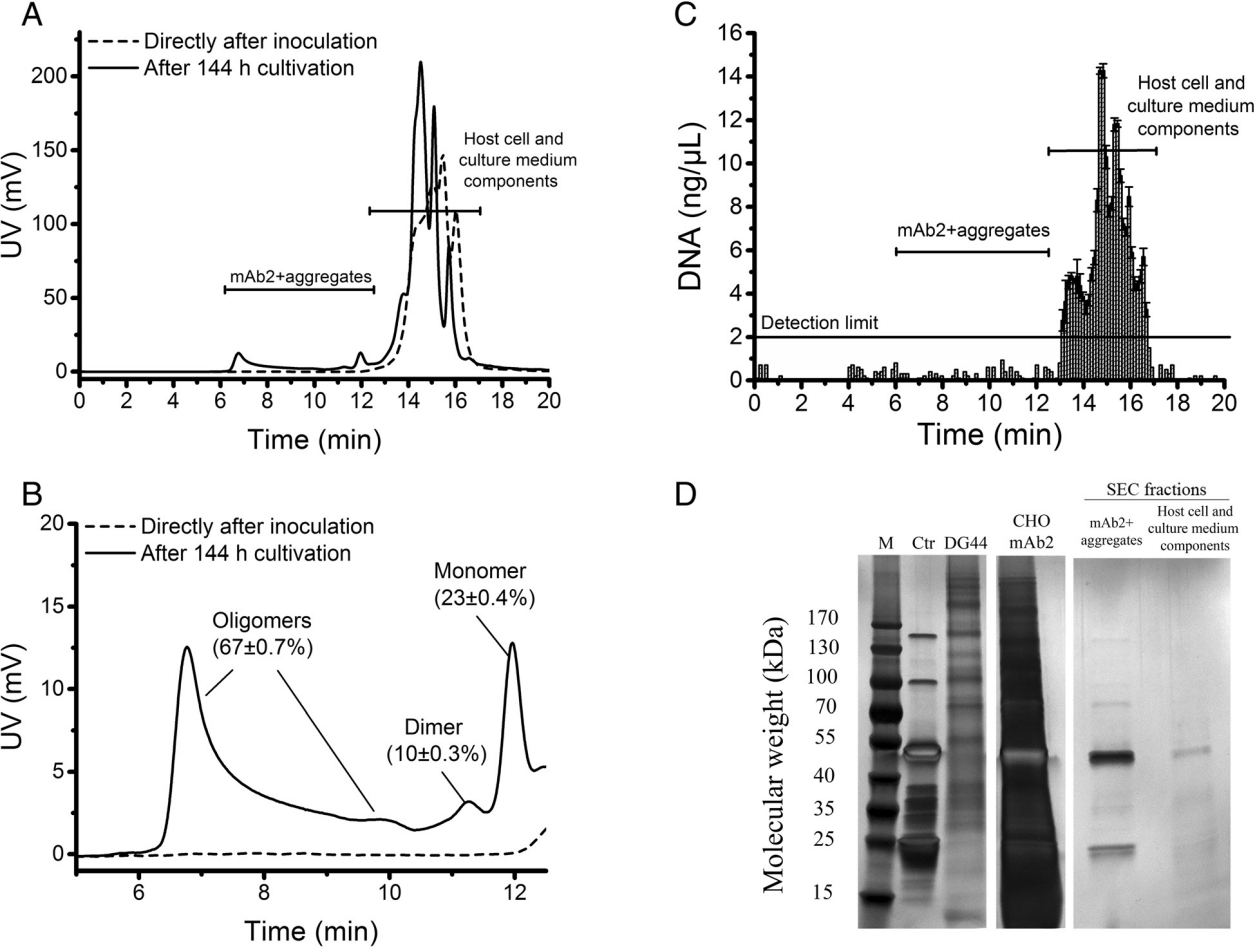
**Analysis of mAb aggregate formation, DNA content, host cell and medium components in the supernatant of a mAb2-producing CHO cell line.** Supernatant was analyzed for mAb aggregate formation directly after inoculation and after 144 h cultivation **(A + B)**. Amount of mAb2 monomer, dimer and oligomers was obtained from biological triplicates ± standard deviation. B is an enlargement of A. DNA content of collected SEC fractions was analyzed using NanoDrop 1000 spectrophotometer **(C)**. For determination of HCPs and culture medium components, SEC fractions were pooled, concentrated and analyzed using SDS-PAGE under reducing conditions with subsequent silver staining **(D)**.

The cultivation showed typical growth, substrate and metabolite concentrations for mAb production (Additional file 2: Figure S1). Directly after inoculation neither monomer nor mAb aggregates were detected (Figure 4A + B). Chromatograms of the SE-HPLC analysis obtained from the supernatant of the three shake flasks showed identical results (Figure 4B). Retention times of the signals obtained immediately after the start of cultivation (14–16 min) corresponded to the elution times obtained in the analysis of the cell-free supernatant from a culture of a non-producing CHO DG44 host cell line (Figure 3H), indicating that the signals were caused by host cell impurities and medium components such as DNA, lipids, HCPs, secreted cellular metabolites and excess nutrients [10,34]. To ensure that these cell culture contaminants do not interfere with quantification of the mAb2 monomer and aggregates in the CHO supernatant, the corresponding chromatography fractions were collected and analyzed for DNA (Figure 4C) and HCPs (Figure 4D). DNA analysis of the collected SE-HPLC fractions revealed that the CHO mAb2 supernatant contained a significant amount of DNA (up to 15 ng μL^−1^). The finally formulated recombinant mAb product has to contain less than 10 ng/dose DNA [35]. Therefore the impurities have to be removed from the cell culture supernatant after harvest using several DSP operations [22]. However, DNA was detected only in the fractions containing host cell and culture medium components and not observed in the SEC fractions used for mAb aggregate quantification. In order to determine the amount of HCPs, the corresponding SEC fractions were pooled, concentrated and separated using reducing SDS-PAGE with subsequent silver staining (Figure 4D). The supernatant of the non-producing CHO cells (DG44) and the supernatant of the mAb2-producing CHO cells (CHO mAb2) both showed a huge amount of signals, indicating presence of HCPs in the supernatant. The strongest bands in the mAb2-producing CHO supernatant were caused by the heavy chain (around 50 kDa) and the light chain (around 25 kDa) of reduced mAb2, also visible in the purified mAb2 control (Ctr). Analysis of the collected SEC fractions revealed that the mAb2 monomer and aggregate fraction contained mainly heavy and light chain, indicating that nearly no HCPs were present. The monomer and aggregate fraction showed a signal at 150 kDa caused by unreduced mAb2 as well as bands between heavy and light chain, which were also visible in the control, whereas the fractions collected from host cell and culture medium components showed additional signals. It is known that mammalian cells secrete HCPs together with the product [36]. Nevertheless, the mAb2 monomer and aggregate fractions collected from the SE-HPLC analysis contained no detectable amounts of DNA and were nearly free of HCP enabling mAb aggregate quantification directly in the supernatant of CHO cells using the SEC columns tested in this work. However, the results also revealed the presence of a large amount of DNA and cell culture components in the supernatant of CHO cell culture and emphasize the importance of the use of appropriate SEC columns for aggregate analysis.

These interfering components may be the reason why analysis of protein aggregation during cell culture was mainly performed after a Protein A capture step in other experiments [11-13,37]. Franco et al. used an analytical gel filtration column with 13 μm bead size, which had a lower resolution than the 3 μm SEC column used in our work. Ho et al. and Jing et al. performed their experiments with the same column type, which showed insufficient separation of mAb monomer from other cell culture signals in our experiments (Figure 1A). This could explain why in those experiments a pre-purification step was necessary to detect aggregates in cell culture samples. Moreover, the amount of HCPs in the harvest product pool is dependent on the host organism, the protein of interest as well as culture and harvest conditions [38]. It is conceivable that the production cell line as well as the non-producing CHO DG44 cell line used in this work did not secrete HCPs, which interfere with mAb monomer and aggregates enabling a smooth quantification. Using our method, mAb monomer and aggregates were detectable directly in the supernatant of CHO mAb producer cells after 144 h of cultivation without interfering with host cell impurities (Figure 4A + B). Quantification of aggregate formation revealed that a huge amount of mAb consisted of aggregates. Comparison of the cell culture samples to retention times of the aggregated mAb standards indicated that the aggregates comprised of dimer and a huge amount of larger oligomers. Figure 4B shows the mean values of monomer, dimer and larger aggregates resulting from all shake flasks. With only 23% ±0.4% remaining monomer, the supernatant after 144 h comprised of 10% ±0.3% dimer and 67% ±0.7% larger oligomers (Figure 4B). This is the first report demonstrating such a high amount of aggregates during CHO cell culture. Franco et al. found about 10% aggregates in all harvest batches of hybridoma cells secreting a monoclonal anti-PSA antibody [13]. Other work using IgG-producing CHO cells reported aggregation rates from 20% up to 30% [7,39]. Ho et al. obtained an aggregate and fragment content of more than 50% by decreasing the light chain to heavy chain (LC:HC) ratio [37]. As mentioned before, most of these approaches measured mAb aggregate formation after a Protein A purification step. This step included loading of the mAbs to the Protein A column under neutral conditions, elution under acidic conditions and neutralization before applying SEC analysis [10,40]. During elution and neutralization the mAbs undergo critical pH values (pH 5 and 6), which induced very large aggregates in our pH-shift experiments (Table 1). With a diameter above 100 nm at pH 5, the mAb2 aggregates induced by pH-shift were by far larger than using the highest NaCl concentration and three cycles of FT. These large aggregates may be filtered during sample preparation or get stuck in the column matrix and could therefore be underrepresented in the subsequent SEC analysis. This would explain the varying amount of aggregates detected in DSP and emphasizes the importance of a more direct method for upstream aggregate analysis. Directly analyzing mAb cell culture samples using high resolution SEC columns does not include shifts to critical pH values. Although the mAbs used in this work were aggregation-prone, our results indicate how much protein may be lost due to formation of HMW species during cell culture. It can be assumed that the HMW species were product-related, since the supernatant of non-producing CHO cells showed no comparable signals (Figure 3H) and the SEC fractions used for mAb aggregate quantification contained neither DNA nor significant amounts of HCP and culture medium components (Figure 4C + D). Furthermore, the large aggregates detected in the supernatant of the mAb2-producing CHO cell line were also visible in the Rayleigh ratio after NaCl- and FT-induction of purified mAb2 (Figure 2). Our results demonstrate that SE-HPLC analysis using a high-resolution column in combination with suitable aggregate standards can be a valuable tool to determine aggregate formation in cell culture, as the method allows quantification of mAb aggregate content within 20 min directly in the cell culture supernatant without falsifying the results by labor-intensive pre-purification of the samples.

## Conclusions

In this study, we have investigated SEC-based quantification of mAb aggregates directly in the supernatant of CHO cell cultures without a pre-purification step. High-resolution SEC columns enabled separation of mAb monomer and aggregates from interfering cell culture components such as DNA, host cell and culture medium contaminants. In order to distinguish between different HMW species formed in cell culture, mAb dimers, tetramers, oligomers and large aggregates with diameter above 100 nm were induced using different stress techniques to be used as aggregate standards. The induced aggregates were still detectable under conditions similar to the cell culture environment. Finally, we demonstrated that the method is applicable to quantify aggregates in the supernatant of a mAb2-producing CHO cell line. The results indicated that over 75% percent of mAb was aggregated proving that aggregate formation may already have its origin in the cell culture and is not only a problem in DSP.

## Additional files

Additional file 1: Table S1. Specifications of SEC columns used in this study. Additional file 2: Figure S1. Cell culture parameters of mAb2-producing CHO cell line. Viable cells and viability (A), glucose and lactate concentration (B), glutamine and ammonium concentration (C) and product formation (D) of a mAb-producing CHO culture. Samples were taken every 24 h over 144 h of cultivation. Viable cells and viability was measured using Cedex XS (Roche). Substrate, metabolite and product concentrations were measured using Konelab Arena 20 (Thermo Scientific).

## Abbreviations

CHO: Chinese Hamster Ovary
DLS: Dynamic light scattering
DSP: Downstream processing
FT: Freeze-thawing
HCP: Host cell protein
HMW: High molecular weight
HPLC: High pressure liquid chromatography
mAbs: Monoclonal antibodies
MALS: Multi angle light scattering
PBS: Phosphate-buffered saline
RI: Refractive index
RT: Room temperature
SEC: Size exclusion chromatography
USP: Upstream processing
UV: Ultraviolet
Zave: Zeta average
