# Supplementary figures and images for: Direct analysis of mAb aggregates in mammalian cell culture supernatant
Source: BMC Biotechnol. 2014 Nov 29;14:99. doi: 10.1186/s12896-014-0099-3 (PMC4256052; doi:10.1186/s12896-014-0099-3)

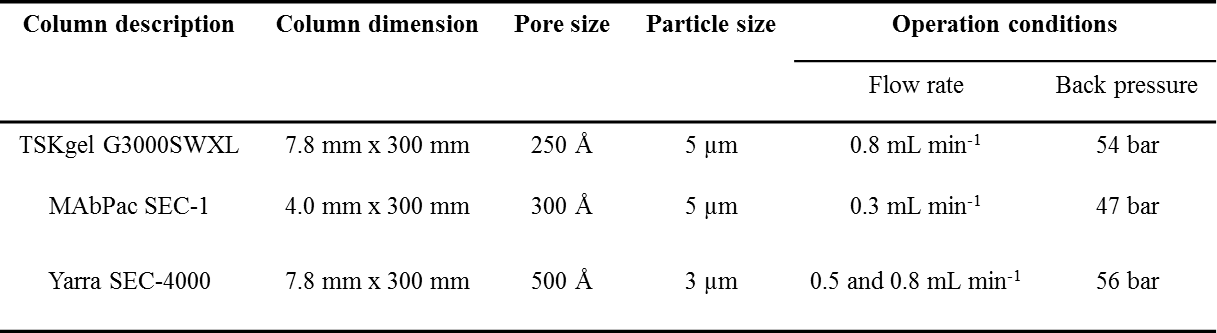

Supplement: Additional file 1: Table S1. — Specifications of SEC columns used in this study. [file 12896_2014_99_MOESM1_ESM.tiff]
